# Immunological and neurotrophic markers of risk status and illness development in high-risk youth: understanding the neurobiological underpinnings of bipolar disorder

**DOI:** 10.1186/2194-7511-2-4

**Published:** 2014-03-31

**Authors:** Anne Duffy, Julie Horrocks, Sarah Doucette, Charles Keown-Stoneman, Paul Grof, Ana Andreazza, L Trevor Young

**Affiliations:** Department of Psychiatry and Hotchkiss Brain Institute (HBI), University of Calgary, TRW Building, 3280 Hospital Drive, NW, Room 4D6, Calgary, AB T2N 4Z6 Canada; Mood Disorders Centre of Ottawa, University of Ottawa Health Services, Ottawa, ON K1N 7B7 Canada; Department of Mathematics and Statistics, University of Guelph, Guelph, ON N1G 2W1 Canada; Department of Community Health & Epidemiology, Dalhousie University, Dalhousie, NS B3H 1V7 Canada; Department of Psychiatry, University of Toronto, Toronto, ON M5T 1R8 Canada

**Keywords:** Bipolar disorder, High-risk, Peripheral markers, Inflammation, Neurotrophic factor, Clinical stages

## Abstract

Bipolar disorder is a highly heritable illness that onsets in adolescence and young adulthood. We examined gene expression (mRNA) and protein levels of candidate immune and neurotrophic markers in well-characterized offspring of bipolar parents in order to identify reliable indicators of illness risk status and the early clinical stages of illness development. We measured mRNA expression and protein levels in candidate immune (TNF-α, IL-1β, IL-10, IFN-δ) and neurotrophic (brain-derived neurotrophic factor (BDNF)) markers from plasma. High-risk offspring were identified from families in which one parent had confirmed bipolar disorder. Control offspring were identified from families in which neither parent met lifetime criteria for a major psychiatric disorder. All parental *Diagnostic and Statistical Manual of Mental Disorders, Fourth Edition* (DSM-IV) diagnoses were based on Schedule for Affective Disorders - Lifetime Version (SADS-L) interviews and blind consensus review. As part of an ongoing study, all offspring were prospectively assessed using KSADS-PL format interviews and diagnoses confirmed on blind consensus review. High-risk offspring had significantly increased IL-6 (*p* = 0.050) and BDNF (*p* = 0.006) protein levels compared to controls. Those high-risk offspring in earlier compared to later clinical stages of illness development had higher IL-6 (*p* = 0.050) and BDNF (*p* = 0.045) protein levels. After adjustments, only differences in BDNF protein levels remained significant. There was a moderating effect of the BDNF genotype on both gene expression and protein levels in high-risk compared to control offspring. The BDNF genotype also moderated the association between clinical stage and gene expression levels in high-risk offspring. These findings provide support for detectable differences in candidate immune and neurotrophic markers in individuals at high risk of developing bipolar disorder and for detectable changes over the clinical stages of illness development. These associations appear to be moderated by genetic variants.

**Electronic supplementary material:**

The online version of this article (doi:10.1186/2194-7511-2-4) contains supplementary material, which is available to authorized users.

## Background

There is convergent evidence from longitudinal studies that bipolar disorder (BD) develops in a series of predictable clinical stages in those at genetic risk (Duffy et al. 2014). A developmental approach to diagnosis is well accepted and useful in other areas of medicine, improving earlier accurate identification, providing the opportunity to develop stage-specific treatments preventing the progression of disease (Scott et al. 2013). In order to refine the clinical staging model and identify novel specific early intervention targets, it is essential to understand the pathophysiological processes associated with the clinical stages of illness development (McGorry et al. 2010).

A large body of evidence supports the association between stress, clinically significant depression, and activation of the immune and neuroendocrine systems (Woiciechowsky et al. 1999; Berk et al. 2011; Gibney and Drexhage 2013), although it still remains unclear if differential reactivity to stress reflects a predisposition or a marker of illness activity or both. The coexistence of elevated circulating levels of pro-inflammatory cytokines and cortisol in patients suffering from acute mood episodes represents an abnormal state, as cytokines typically negatively feedback to decrease release of hypothalamic corticotrophin-releasing factor which in turn decreases cortisol levels (Connor and Leonard 1998). However, clinical depression has been associated with a failure of the normal inhibitory feedback pathway of cortisol on cytokine secretion, in at least a subset of patients (Woiciechowsky et al. 1999). Specifically, in patients with established illness, hypothalamic-pituitary-adrenal (HPA) activation is associated with acute exacerbations as a state marker (Deshauer et al. 1999), and recent evidence suggests that this defect in inhibitory feedback resulting in both immune and HPA axis activation may be a useful trait marker in high-risk individuals (Duffy et al. 2012; Halligan et al. 2007; Ellenbogen et al. 2011).

Specifically, Padmos et al. (2008) reported increased mRNA expression in a series of genes relevant to inflammation in BD adults and their offspring. Increased expression was especially evident in remitted offspring with a lifetime history of depression or in those who went on to develop a depressive episode shortly after sampling. As summarized by Berk et al. (2011), an increase in pro-inflammatory markers is associated with acute BD episodes of both depressive and manic polarity. Interestingly, with increasing illness episodes, there appear to be a persistent perturbation in the balance of pro- and anti-inflammatory cytokines (Kauer-Sant'Anna et al. 2009) and a decrease in brain-derived neurotrophic factor (BDNF) suggestive of a neuroprogressive course. Effective stabilization with lithium has been associated with re-establishing the balance between pro- and anti-inflammatory markers and with neuroprotection (Berk et al. 2011; Kapczinski et al. 2008; Hajek et al. 2013). Collectively, the findings suggest that at least in a subset of individuals, subtle perturbations in neuroimmune markers may correlate with the early clinical stages of illness development in high-risk individuals and serve as an important early intervention target (Raison et al. 2010; Maes et al. 2012).

In this paper, we present findings from a cross-sectional study of DNA polymorphisms, mRNA expression, and protein levels in candidate immune system (TNF-α, IL-1β, IL-10, IFN-δ) and neurotrophic (BDNF) markers from plasma collected in prospectively assessed high-risk offspring of well-characterized BD parents and from well offspring of psychiatrically unaffected parents (controls). The markers were selected based on the most robust findings in the literature (Padmos et al. 2008; Frey et al. 2013). We tested the hypothesis that high-risk offspring would show differences in the gene expression and protein levels of candidate markers compared to controls. Secondly, we explored differences in gene expression and protein levels associated with early compared to later stage illness development in high-risk offspring using a previously published staging model (Duffy et al. 2010,2014). Finally, we explored whether genetic variants of each candidate marker influenced these associations.

## Results

### Sample description

The sample included 19 high-risk and 16 control offspring with a mean age at sample collection of 21.89 (standard deviation (SD) 4.04) and 20.44 (SD 2.61) years, respectively. High-risk offspring had a mean GAF score of 86.11 (SD 8.12) and the control mean GAF score was 89.19 (SD 5.88) at the time of sampling. There were no significant differences in SES, age, or sex between high-risk and control offspring (all *p* > 0.05). The distribution of variants for each of the five markers studied was similar between high-risk and control groups. As would be expected in a high-risk cohort early in the developmental course of illness, the GAF scores were comparable to controls.

### Clinical characteristics

For those high-risk offspring meeting operational criteria for later stage illness development (major depression or BD lifetime), the clinical characteristics are presented in Additional file 1: Table S1. High-risk offspring who met *Diagnostic and Statistical Manual of Mental Disorders, Fourth Edition* (DSM-IV) criteria for a major mood disorder had a mean illness onset, as defined by age of first meeting full diagnostic criteria for a major mood episode, of 16.37 years (SD 4.86). The duration (weeks) of acute illness episodes before sampling in this subgroup of remitted high-risk offspring was 41.57 weeks (SD 25.12). Only two high-risk offspring were taking mood stabilizers and no offspring was taking any other psychotropic at the time of blood draw, and only four had ever been exposed to a therapeutic trial of any psychotropic medication.

### Gene expression (mRNA) and protein levels of candidate genes

**Figure 1:**
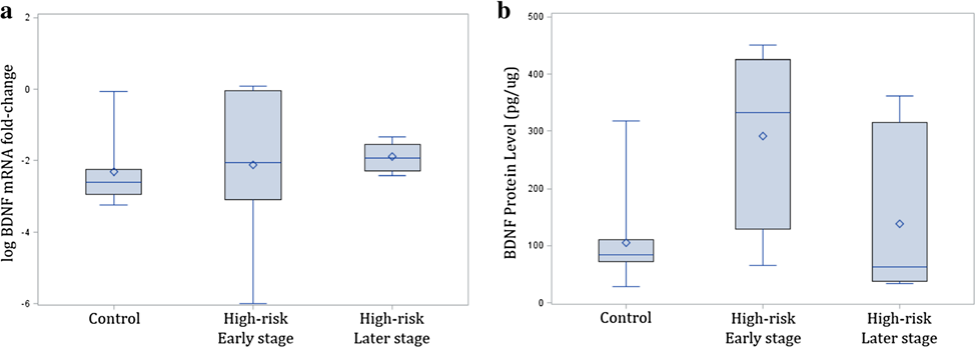
**BDNF log mRNA expression (a) and protein levels (b) in control and high-risk early and later stages of illness development.**

**Figure 2:**
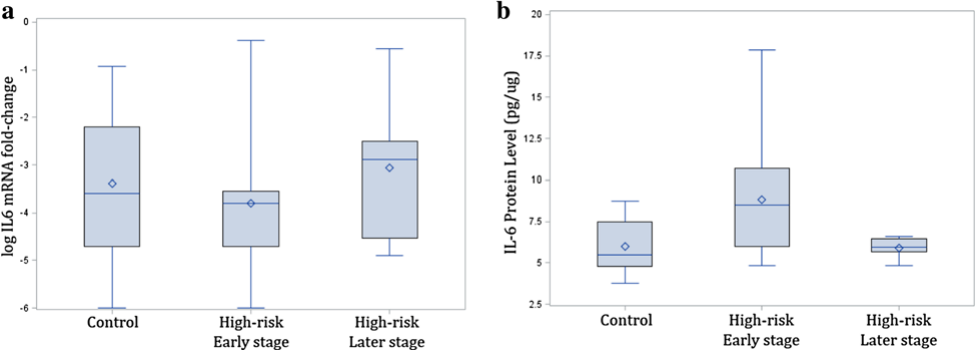
**IL-6 log mRNA expression (a) and protein levels (b) in control and high-risk early and later stages of illness development.**

As presented in Table 1, using unadjusted *t* tests for comparing two independent groups, there were no significant differences in the mRNA expression levels of candidate genes between high-risk and control offspring or between high-risk offspring in the early compared to those in later clinical stages of illness development; however, compared to controls, high-risk offspring had higher IL-6 (*p* = 0.050) and BDNF (*p* = 0.006) protein levels. In addition, earlier stage high-risk offspring had higher IL-6 and BDNF protein levels compared to high-risk offspring in the later stages of BD development (*p* = 0.050; *p* = 0.045) (see Figures 1 and 2 for box plots).

**Table 1:** **Unadjusted mRNA expression and protein levels of candidate markers between groups**

		High-risk vs. control offspring					High-risk early vs. later clinical stage				
		Control n = 16		High-risk n = 19		***p*** value^a^	Early n = 12		Later n = 7		***p*** value^b^
		Mean	SD	Mean	SD		Mean	SD	Mean	SD	
BDNF	mRNA	0.1753	(0.2616)	0.2737	(0.3525)	0.5410	0.3464	(0.4403)	0.1593	(0.0635)	0.7346
	Protein	105.13	(68.85)	234.83	(162.69)	0.0056	290.96	(152.59)	138.60	(140.03)	0.0453
TNF	mRNA	0.2290	(0.2615)	0.4759	(0.8113)	0.2485	0.6521	(1.012)	0.1991	(0.1089)	0.4747
	Protein	55.53	(20.07)	54.33	(12.28)	0.8292	56.22	(12.46)	51.09	(12.16)	0.3951
IFN	mRNA	0.0388	(0.0688)	0.0615	(0.0658)	0.0803	0.0584	(0.0795)	0.0663	(0.0410)	0.2447
	Protein	81.56	(26.80)	70.99	(28.70)	0.2715	63.36	(18.93)	84.09	(38.70)	0.1324
IL-6	mRNA	0.0740	(0.1063)	0.0966	(0.1956)	0.8091	0.0846	(0.2014)	0.1156	(0.2005)	0.3192
	Protein	5.98	(1.62)	7.77	(3.18)	0.0505	8.84	(3.60)	5.92	(0.59)	0.0502
IL-10	mRNA	0.0549	(0.0521)	0.0836	(0.0779)	0.7390	0.0683	(0.0620)	0.1076	(0.0984)	0.7219
	Protein	16.16	(8.82)	19.36	(6.04)	0.2132	19.11	(5.79)	19.78	(6.90)	0.8243

mRNA means and SD are presented as fold-change; protein levels are measured in picograms per microgram. ^a^ *p* value for *t* test comparing HR to control; mRNA is log-transformed; ^b^ *p* value for *t* test comparing early stage to late stage; mRNA is log-transformed.

Using a linear regression model adjusting for sex, age, GAF, and SES, there was a significant difference between high-risk and control offspring in BDNF protein levels (*p* = 0.026). It was estimated that high-risk offspring had 136.67 pg/μg higher mean BDNF protein levels than controls. After adjustment, there was no evidence of differences between high-risk and control offspring for any other proteins examined (*p*_(TNF)_ = 0.377, *p*_(IFN)_ = 0.226, *p*_(IL-6)_ = 0.172, *p*_(IL-10)_ = 0.547) or mRNA expression levels (*p*_(BDNF)_ = 0.274, *p*_(TNF)_ = 0.229, *p*_(IFN)_ = 0.115, *p*_(IL-6)_ = 0.184, *p*_(IL-10)_ = 0.731).

When comparing high-risk offspring in early clinical stages to high-risk offspring in later clinical stages after adjustment, there were no differences in protein levels (*p*_(BDNF)_ = 0.1094, *p*_(TNF)_ = 0.412, *p*_(IFN)_ = 0.329, *p*_(IL-6)_ = 0.151, *p*_(IL-10)_ = 0.929) or mRNA expression (*p*_(BDNF)_ = 0.691, *p*_(TNF)_ = 0.376, *p*_(IFN)_ = 0.321, *p*_(IL-6)_ = 0.840, *p*_(IL-10)_ = 0.796) in all markers examined.

#### Moderating effects of genetic variants on high-risk status for gene expression and protein levels

As indicated in Table 2, for log mRNA expression, there was a significant interaction between the BDNF genotype and high-risk status after adjustments (*p* = 0.028). In particular, among those with the VAL/VAL genotype, the high-risk group (*n* = 9) had higher log mRNA expression levels than the control group (*n* = 10) (*p* = 0.024). In the high-risk group, those with the VAL/VAL genotype had higher log mRNA expression than those carrying a MET allele (*n* = 8) (*p* = 0.003) (see Additional file 1: Table S2a for pair-wise comparisons)

**Table 2:** **Interaction between genotype and high-risk status and genotype and clinical stage**

	Genotype × high-risk status predicting log mRNA expression	Genotype × high-risk status predicting protein levels	Genotype × clinical stage predicting log mRNA expression	Genotype × clinical stage predicting protein levels
	***p*** value	***p*** value	***p*** value	***p*** value
BDNF	0.0276	0.0403	0.0634	0.4785
TNF	0.1701	0.6675	0.1438	0.5853
IFN	0.3378	0.9346	0.7294	0.9805
IL-6	0.4774	0.1593	0.7182	0.0688
IL-10	0.5449	0.3154	0.2591	0.3105

log mRNA expression presented as log fold-change; protein levels are measured in picograms per microgram. All values are adjusted for sex, age, SES, and GAF.

**Figure 3:**
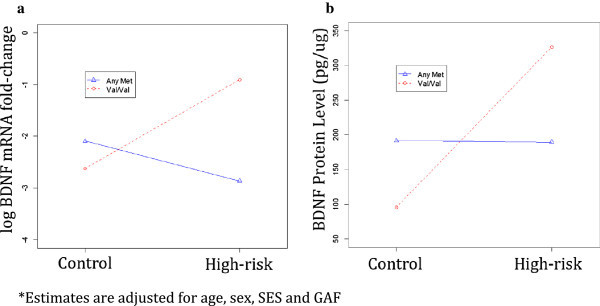
**BDNF log mRNA expression (a) and protein levels (b) in high-risk and control offspring.** MET genotype (*blue lines*) and VAL/VAL genotype (*red lines*)*.

There was also a significant interaction for protein levels between the BDNF genotype and high-risk status after adjustments (*p* = 0.040). In particular, among those with the VAL/VAL genotype, the high-risk group had higher protein levels than the control group (*p* = 0.004). In the high-risk group, those with the VAL/VAL genotype had higher protein levels than MET carriers (*p* = 0.030) (see Additional file 1: Table S2b for pair-wise comparisons and Figure 3).

For IL-6, high-risk offspring with the GG variant (*n* = 14) had higher protein levels than control offspring (*n* = 9) after adjustments (*p* = 0.035). However, the global *F* test for interaction between the IL-6 genotype and high-risk status (providing some protection for multiple comparisons) was not significant (*p* = 0.159) (see Table 2).

#### Moderating effects of genetic variants on stage of illness development for gene expression and protein levels

As indicated in Table 2, for log mRNA expression levels, there was marginal significance of an interaction between the BDNF genotype and illness development stage after adjustments (*p* = 0.063). In the early stage of illness development, those high-risk offspring with a VAL/VAL genotype (*n* = 6) had higher mRNA expression levels than those high-risk offspring who were MET carriers (*n* = 4) (*p* = 0.003). Among those high-risk offspring who were MET carriers, those in the later stages of illness development (*n* = 4) had higher mRNA expression than those in the earlier stages (*n* = 4) (*p* = 0.038) (see Additional file 1: Table S2c for pair-wise comparisons).

For TNF, among those high-risk offspring in the early stages of illness development, those with a GG genotype (*n* = 8) had higher log mRNA expression than those with an AG genotype (*n* = 2) after adjustments (*p* = 0.016). Also, among those high-risk offspring with the GG genotype, those in the early stages of illness development had higher log mRNA expression than those in the later stages (*n* = 6) after adjustments (*p* = 0.024). However, the overall interaction test was not significant (*p* = 0.144).

As indicated in Table 2, for protein levels, there was marginal significance of an interaction between the IL-6 genotype and illness stage after adjustments (*p* = 0.069). Among those with a GG genotype, high-risk offspring in the earlier stages of illness development (*n* = 6) had higher protein levels than those in the later stages of illness development (*n* = 1) (*p* = 0.057). There was no evidence of pair-wise differences in BDNF protein levels (see Additional file 1: Table S2d for pair-wise comparisons).

In high-risk offspring in the later stages of illness development, those with an AG genotype (*n* = 4) had significantly higher IL-10 protein levels than those with an AA genotype (*n* = 3) after adjustments (*p* = 0.046). However the overall interaction test was not significant (*p* = 0.310) (Table 2).

## Discussion

There has been a substantial literature linking mood disorders with altered immune functioning, in at least a subset of patients. Acute mood episodes are associated with an inflammatory bias, and effective antidepressant and mood-stabilizing treatments have been shown to restore the balance between pro- and anti-inflammatory mediators (Raison and Miller 2011). Patients with acute mood episodes are at increased risk of developing systemic inflammatory illnesses, and patients with inflammatory-based illnesses have a higher risk of developing mood disorders (Connor and Leonard 1998). Furthermore, there have been recent reports of a shift in the balance of immune and neurotrophic mediators over the course of established BD, suggesting the development of a more severe dysregulation in the immune system and reduction in neurotrophic factors in patients with recurrent episodes of illness, poor remission, and a high burden of illness (Berk et al. 2011; Kauer-Sant'Anna et al. 2009).

Interestingly, there is accruing evidence that increased pro-inflammatory markers and related HPA axis dysregulation are detectable early in the developing course of bipolar illness in genetically high-risk adolescents, even before the onset of the first major mood episodes (Duffy et al. 2012). In this investigation, we studied DNA polymorphisms, gene expression, and protein levels of pro-inflammatory mediators IL-1β, IL-6, TNF-α, and IFN-δ, anti-inflammatory mediator IL-10, and BDNF in lymphocytes and plasma collected from offspring at genetic high risk compared to low-risk offspring. We divided the high-risk offspring into two groups based on their place in a clinical staging model describing the development of bipolar illness (Duffy et al. 2010).

We found differences in protein levels in candidate genes related to inflammation and neuroprotection between young people at high risk of developing BD in comparison to low-risk controls. Specifically, both IL-6 and BDNF protein levels were higher in high-risk compared to low-risk offspring. We also found that protein levels of candidate genes changed over the clinical stages of illness development. Specifically, we found a significant difference in IL-6 and BDNF protein levels between high-risk offspring in the early compared to the later stages of illness development. While only differences between high-risk and control offspring for BDNF protein levels remained significant after adjustment, this was likely due to the small sample size. These findings support the hypothesis that there are detectable differences in immune and neurotrophic markers in high-risk individuals and that these change over the course of illness development. This hypothesis requires systematic longitudinal investigation within high-risk subjects over the early clinical stages of illness development.

We also found that the genotype of high-risk individuals significantly influences the association between high-risk status and clinical stage of illness development for both gene expression and protein levels. Specifically, in this study, there was a significant interaction between the BDNF genotype and high-risk status for both gene expression and protein levels. The VAL/VAL genotype has been associated with early-onset mood disorders (Strauss et al. 2004). In a recent study, Goodyer et al. (2010) found an interaction between elevated morning cortisol and presence of the VAL/VAL genotype in predicting increased risk of depression in high-risk adolescents, underscoring the importance of an integrative perspective when interpreting the moderating effects of gene variants on illness outcome.

Interestingly, there were also significant interactions found in this study between the clinical stage of illness development and BDNF and IL-6 genotypes, for both gene expression and protein levels, respectively. This observation is consistent with other reports in patients with well-established BD of an association between clinical course and changes in the balance of pro-inflammatory mediators and neurotrophic factors (Kauer-Sant'Anna et al. 2009). The key point from our findings is that changes in biomarkers seem to occur over the course of illness development, as well as in association with burden of illness effects.

The major limitation of this study is the small number of high-risk and comparison offspring, as well as the cross-sectional study design. Therefore, findings should be viewed as preliminary and hypothesis generating. However, all subjects included in this study have been repeatedly prospectively assessed in clinical interviews, and their parents have been studied longitudinally to ensure stability of diagnosis (i.e., high-risk status). Furthermore, there was minimal confounding with exposure to medication. Undoubtedly, a longitudinal design tracking changes in candidate biomarkers within high-risk subjects over illness development would be a much more powerful approach to understand the pathophysiology underlying illness development and to validate and refine the clinical stages. However, given the limitations discussed, this study does provide evidence suggesting that there are identifiable differences in mRNA expression and protein levels in candidate immune and neurotrophic peripheral markers in high-risk offspring, which are moderated by genetic variants. Also, there appear to be changes in candidate biological markers across the clinical stages of illness development. However, we emphasize that these findings are preliminary and require systematic longitudinal study in a larger well-characterized high-risk cohort.

## Conclusions

It is increasingly acknowledged that an important way forward is to invest in studies aimed at identifying genetically sensitive biological makers that relate to etiological processes, treatment response, and longer-term outcomes (Goodyer et al. 2010). The success of such research is predicated on starting with well-characterized high-risk samples to reduce heterogeneity. In order to provide an integrated view, it would be prudent to include a number of associated physiological makers indexing interactive immune and neuroendocrine pathways. In this way, genetically sensitive pathways associated with the etiology of BD can be separated from the burden of illness effects related to recurrent episodes, psychotic symptoms, treatment, and medical and psychiatric complications. This research would lead to a comprehensive working model of how BD develops in genetically at-risk individuals and refine the clinical staging model, while identifying novel stage-specific targets for intervention and prevention of illness progression.

## Methods

### Subjects

In compliance with the Helsinki Declaration, this research was approved by local research ethics boards in Ottawa, Halifax, and Calgary.

In this study, we recruited 19 high-risk and 16 comparison offspring from families participating in an ongoing longitudinal study describing the early natural history of BD (MOP 102761) (Duffy et al. 2009,2010). Specifically, we enrolled consenting adolescent and young adult offspring from parents with BD based on Schedule for Affective Disorders - Lifetime Version (SADS-L) interview conducted by a research psychiatrist and final DSM-IV diagnosis confirmed by blind consensus review using all available clinical information (other parents no lifetime history of major psychiatric disorders). Consenting comparison offspring were recruited from families in which neither parent met DSM-IV criteria for a lifetime major psychiatric disorder (psychotic, mood, substance use disorders) on the basis of SADS-L interviews and blind consensus diagnostic review.

As part of the ongoing longitudinal study, all offspring were clinically assessed annually using KSADS-PL format interviews conducted by a psychiatrist. Final DSM-IV diagnoses were confirmed on the basis of blind consensus review including two additional experienced clinician researchers (one being a senior research psychiatrist) using all available clinical information. At the time of blood sampling, all subjects were at their best level of functioning (well or in remission) and completed the Beck Depression Inventory (BDI) (Beck and Beamesderfer 1974) and the clinician completed the Global Assessment of Functioning Scale (GAF) (Hall 1995). Subjects were excluded from this study if they were acutely psychiatrically or medically ill, abusing substances, or taking anti-inflammatory medications (i.e., prednisone, ASA, steroid inhalers) within 6 months of sampling.

We used a novel clinical staging model to subdivide high-risk subjects into those in the early or later stages of bipolar illness development (see Duffy et al. 2010,2014). Briefly, high-risk offspring were classified as follows: *stage 0* if they were clinically well; *stage 1* if they met lifetime criteria for non-specific disorders (ADHD, anxiety, sleep); *stage 2* if they met lifetime criteria for sub-affective mood disorders (dysthymia, depression NOS, adjustment disorder with anxiety and depressive symptoms); *stage 3* if they met lifetime criteria for major depressive disorder (single episode or recurrent); and *stage 4* if they met lifetime criteria for a diagnosable BD (BDNOS, BD I or II, schizoaffective-BD). For this analysis, we defined *early stage* illness as stages 0 to 2 and *later stage* illness development as stages 3 to 4, given that BD most often debuts as major depressive episodes (Duffy 2010).

### Biochemical assays

#### Blood collection

Twenty milliliters of blood was drawn from each subject by venipuncture into a free-anticoagulant vacuum tube. Ficoll-Paque PLUS (71-7167-00 AG, GE Healthcare, Uppsala, Sweden) was used to extract white blood cells. Briefly, 4 ml of diluted blood (1:1) was carefully layered on 4 ml of Ficoll-Paque followed by centrifugation at 400 *g* for 40 min. First, plasma was removed and then the lymphocyte ring was carefully extracted. Lymphocytes were washed with balanced salt solution three times. Plasma and lymphocytes were kept frozen at −80°C until assayed. Lymphocytes samples were used to extract DNA and RNA for genotyping and mRNA expression studies, and plasma was used to evaluate the protein levels. All samples were de-identified and coded at the time of sample collection, and the laboratory remained blind to the study group and family affiliation.

#### Gene expression

For the gene expression study, a two-step reverse transcription polymerase chain reaction (RT-PCR) was performed. Miniprep columns were used to isolate RNA from lymphocytes according to the manufacturer's protocol (RNeasy® by Qiagen, Venlo, The Netherlands). RNA concentrations were determined using a NanoDrop™ 1000 Spectrophotometer System® (Thermo Scientific, Wilmington, DE, USA); 0.5 μg of total RNA was reverse-transcribed to generate high-fidelity cDNA employing a kit from the same manufacturer (QuantiTect® Reverse Transcription Kit). Assuming a 1:1 conversion of RNA to cDNA, 15 ng of cDNA was used to perform qPCR with a commercial mix (QuantiFast® SYBR® Green PCR Kit by Qiagen). All primers were designed spanning an exon/exon boundary to eliminate amplification of contaminating genomic DNA (QuantiTect® Primers by Qiagen). Thermal cycling conditions were as follows: 1 cycle of 5 min at 95°C (hot start step), 40 cycles of 10 s at 95°C followed by 30 s at 60°C, and finally a melting curve ranging from 65°C to 95°C. Real-time RT-PCR amplifications were run on a CFX 96™ Real-Time PCR Detection System (Bio-Rad, Hercules, CA, USA) within a 25-μl final volume reaction. All reactions were performed in duplicate per experiment, and β-actin was included in all experiments as an endogenous control. Data were expressed as cycle threshold (CT) values being normalized against β-actin. Gene expression results were calculated using the 2^−∆∆CT^ relative quantification method. The primer IDs used were as follows: HsBDNF1SG, HsIL101SG, HsTNF3SG, HsIFNG1SG, HsIL61SG.

#### Protein levels

Protein levels of IL-10, IL-6, TNF-alpha, IFN, and BDNF were assessed by sandwich ELISA, using a commercial kit according to the manufacturer's instructions (Cedarlane (Burlington, Canada): IL-10 #CL76130, IL-6 #CL76126K, TNF-alpha #CL76145K, IFN #CL76120K; Millipore (Billerica, MA, USA): BDNF #CYT306).

#### DNA genotyping

Genomic DNA was extracted from lymphocyte samples using a Miniprep column system (GenElute^TM^ Mammalian Genomic DNA Miniprep Kit by Sigma, St. Louis, MO, USA). DNA concentrations were determined applying the same system used previously for the gene expression experiment.

#### Amplification: 20 ng of DNA

Twenty nanograms of DNA was amplified for each sample for seven variants across six using TaqMan®. The samples were amplified as per manufacturer's directions in a total volume of 10 μl. For each variant, six no template control (NTC) samples were run simultaneously for quality control purposes. Post-amplification products were visualized on the ViiA™ 7 Real-Time PCR System, and genotype calls were assigned manually.

### Statistical approach

Unadjusted differences between groups were tested using *t* tests and, where noted, Fisher's exact tests. Linear regression models were used to test for differences between groups and for interactions between group and DNA variant, after adjustment for sex, age, socio-economic status (SES), and GAF. When conducting *t* tests and linear regression analysis, mRNA expression levels were log-transformed in order to satisfy the normality assumption. All DNA variant allele combinations were treated as categorical with three levels for each marker, with the exception of BDNF, which was treated as a binary variable representing the presence or absence of a MET DNA variant. No adjustments for multiple comparisons were made. Analyses were conducted using SAS version 9.3.

#### Supplementary tables

To elucidate interactions between group and DNA variant, *t* tests for specific contrasts (pair-wise differences) in the least squares means were performed.

## Electronic supplementary material

Additional file 1: **Supplementary tables. Table S1.** Clinical variables in high-risk offspring with a lifetime mood disorder (clinical stage 3-4). **Table S2a.** Differences in BDNF log mRNA expression between high-risk and control offspring with and without the MET variant. **Table S2b.** Differences in BDNF protein levels between high-risk and control offspring with and without the MET variant. **Table S2c.** Differences in BDNF log mRNA expression between early and late stage illness in high-risk offspring with and without the MET variant. **Table S2d.** Differences in BDNF protein levels between early and late stage illness in high-risk offspring with and without the MET variant. (DOC 71 KB)

## Abbreviations

ADHD: attention deficit hyperactivity disorder
BD: bipolar disorder
BDI: Beck Depression Inventory
BDNF: brain-derived neurotrophic factor
CT: cycle threshold
DSM-IV: Diagnostic and Statistical Manual of Mental Disorders, Fourth Edition
GAF: Global Assessment of Functioning Scale
HPA: hypothalamic-pituitary-adrenal
IFN: interferon
IL-6: interleukin-6
IL-10: interleukin-10
KSADS-PL: Kiddie Schedule for Affective Disorders - Lifetime Version
NOS: not otherwise specified
NTC: no template control
SADS-L: Schedule for Affective Disorders - Lifetime Version
SES: socio-economic status
TNF-α: tumor necrosis factor-alpha.
