# Supplementary material for: Immunological and neurotrophic markers of risk status and illness development in high-risk youth: understanding the neurobiological underpinnings of bipolar disorder
Source: Int J Bipolar Disord. 2014 Mar 31;2:4. doi: 10.1186/2194-7511-2-4 (PMC4447739; doi:10.1186/2194-7511-2-4)
Supplement: Supplementary file 1 — Additional file 1: Supplementary tables. Table S1. Clinical variables in high-risk offspring with a lifetime mood disorder (clinical stage 3-4). Table S2a. Differences in BDNF log mRNA expression between high-risk and control offspring with and without the MET variant. Table S2b. Differences in BDNF protein levels between high-risk and control offspring with and without the MET variant. Table S2c. Differences in BDNF log mRNA expression between early and late stage illness in high-risk offspring with and without the MET variant. Table S2d. Differences in BDNF protein levels between early and late stage illness in high-risk offspring with and without the MET variant. (DOC 71 KB) [file 40345_2014_29_MOESM1_ESM.doc]

**Supplementary Table 1S.**

Clinical variables in high-risk offspring with a lifetime mood disorder

(clinical stage 3-4)

|  | Later Stage, n=7 | |
| --- | --- | --- |
|  | Mean (SD) | |
| GAF Score | 83.57 | (10.29) |
| BDI Score | 4.80 | (4.49) |
| Age Onset Mood Disorder | 16.37 | (4.86) |
| # Prior Depressive Episodes | 3 | (1.46) |
| # Prior Activated Episodes | 1 | (0.79) |
| Duration Mood Disorder (Weeks)^a^ | 41.57 | (25.12) |
|  | n | (%) |
| Lifetime Major Depressive Disorder | 4 | (57.14) |
| Lifetime Bipolar Disorder | 3 | (42.86) |
| Lifetime Psychotic Features | 2 | (28.57) |

GAF: Global assessment of functioning; BDI: Beck Depression Inventory

^a^Duration of mood disorder is from the index episode to sampling time

**Supplementary Table 2Sa.**

Differences in BDNF log mRNA expression between high-risk and control offspring with and without the MET variant

| **Group** | **DNA**  **Variant** | **Mean** | **Group** | **DNA**  **Variant** | **Mean** | ***p-value*** |
| --- | --- | --- | --- | --- | --- | --- |
| Control | no MET | -2.6248 | Control | MET | -2.0943 | 0.5171 |
| Control | no MET | -2.6248 | High-risk | no MET | -0.9118 | 0.0242 |
| Control | MET | -2.0943 | High-risk | MET | -2.8631 | 0.3182 |
| High-risk | no MET | -0.9118 | High-risk | MET | -2.8631 | 0.0031 |

Legend: significant findings are highlighted, when comparing groups the estimated higher group is highlighted in orange**.**

All values are adjusted for sex, age, SES and GAF

**Supplementary Table 2Sb.**

Differences in BDNF protein levels between high-risk and control offspring with and without the MET variant

| **Group** | **DNA**  **Variant** | **Mean** | **Group** | **DNA**  **Variant** | **Mean** | ***p-value*** |
| --- | --- | --- | --- | --- | --- | --- |
| Control | no MET | 96.14 | Control | MET | 191.55 | 0.2574 |
| Control | no MET | 96.14 | High-risk | no MET | 326.45 | 0.0044 |
| Control | MET | 191.55 | High-risk | MET | 189.41 | 0.9779 |
| High-risk | no MET | 326.45 | High-risk | MET | 189.41 | 0.0296 |

Legend: significant findings are highlighted, when comparing groups the estimated higher group is highlighted in orange**.**

All values are adjusted for sex, age, SES and GAF

**Supplementary Table 2Sc.**

Differences in BDNF log mRNA expression between early and late stage illness in high-risk offspring with and without the MET variant

| **Group** | **DNA**  **Variant** | **Mean** | **Group** | **DNA**  **Variant** | **Mean** | ***p-value*** |
| --- | --- | --- | --- | --- | --- | --- |
| Early | no MET | -0.6279 | Early | MET | -3.7079 | 0.0032 |
| Early | no MET | -0.6279 | Late | no MET | -1.3629 | 0.4552 |
| Early | MET | -3.7079 | Late | MET | -1.6123 | 0.0379 |
| Late | no MET | -1.3629 | Late | MET | -1.6123 | 0.8217 |

Legend: significant findings are highlighted, when comparing groups the estimated higher group is highlighted in orange**.**

All values are adjusted for sex, age, SES and GAF

**Supplementary Table 2Sd.**

Differences in BDNF protein levels between early and late stage illness in high-risk offspring with and without the MET variant

| **Group** | **DNA**  **Variant** | **Mean** | **Group** | **DNA**  **Variant** | **Mean** | ***p-value*** |
| --- | --- | --- | --- | --- | --- | --- |
| Early | no MET | 312.90 | Early | MET | 241.50 | 0.2705 |
| Early | no MET | 312.90 | Late | no MET | 290.36 | 0.7753 |
| Early | MET | 241.50 | Late | MET | 139.33 | 0.1757 |
| Late | no MET | 290.36 | Late | MET | 139.33 | 0.1218 |

Legend: significant findings are highlighted, when comparing groups the estimated higher group is highlighted in orange**.**

All values are adjusted for sex, age, SES and GAF
